# Spoken discourse in episodic autobiographical and verbal short-term memory in Chinese people with dementia: the roles of global coherence and informativeness

**DOI:** 10.3389/fpsyg.2023.1124477

**Published:** 2023-10-31

**Authors:** Anthony Pak-Hin Kong, Ryan Tsz Him Cheung, Gloria H. Y. Wong, Jacky C. P. Choy, Ruizhi Dai, Aimee Spector

**Affiliations:** ^1^Academic Unit of Human Communication, Development, and Information Sciences, The University of Hong Kong, Hong Kong, Hong Kong SAR, China; ^2^Aphasia Research and Therapy (ART) Laboratory, The University of Hong Kong, Hong Kong, Hong Kong SAR, China; ^3^Department of Social Work and Social Administration, The University of Hong Kong, Hong Kong, Hong Kong SAR, China; ^4^Department of Clinical, Educational and Health Psychology, University College London, London, United Kingdom; ^5^Department of Health Service and Population Research, Institute of Psychiatry, Psychology and Neuroscience (IoPPN), King’s College London, London, United Kingdom; ^6^Department of Psychology, Guangzhou University, Guangzhou, China

**Keywords:** dementia, spoken discourse, personal narratives, sequential picture description, episodic autobiographical memory, verbal short-term memory, global coherence, informativeness

## Abstract

**Introduction:**

Memory and discourse production are closely related in healthy populations. A few studies in people with amnestic mild cognitive impairment and people with dementia (PWD) suggested similar links, although empirical evidence is insufficient to inform emerging intervention design and natural language processing research. Fine-grained discourse assessment is needed to understand their complex relationship in PWD.

**Methods:**

Spoken samples from 104 PWD were elicited using personal narrative and sequential picture description and assessed using Main Concept Analysis and other content-based analytic methods. Discourse and memory performance data were analyzed in bivariate correlation and linear multiple regression models to determine the relationship between discourse production and episodic autobiographical memory and verbal short-term memory (vSTM).

**Results:**

Global coherence was a significant predictor of episodic autobiographical memory, explaining over half of the variance. Both episodic autobiographical memory and vSTM were positively correlated with global coherence and informativeness, and negatively with empty speech indices.

**Discussion:**

Coherence in personal narrative may be supported by episodic autobiographical memory and vice versa, suggesting potential mechanism of interventions targeting personhood through conversation. Indices of global coherence, informativeness, and empty speech can be used as markers of memory functions in PWD.

## 1. Introduction

Memory impairments underlie a range of cognitive and functional deficits in people with dementia (PWD). Impairment in episodic memory is the clinical hallmark for Alzheimer’s disease (AD) and other types of dementia (Arlt, 2013; Economou et al., 2016). More specifically, autobiographical memory decline is linked to sense of self and identity (Caddell and Clare, 2010; El Haj et al., 2015), with PWD demonstrating impoverished self-representation (Ben Malek et al., 2019). Reduced details in autobiographical memory can also be noted in very early dementia before episodic memory impairments can be detected using standardized memory tests (Lindsay et al., 2021). Episodic autobiographical memory is the memory for specific events from one’s own past. Greater richness in episodic autobiographical memory supports better subjective re-experiencing of the past, including emotions and thoughts (Irish et al., 2018) (for a review, see Allen et al., 2018). On the other hand, deficits in verbal short-term memory (vSTM), the ability to actively maintain verbal information mentally for brief periods, have also been widely reported (Ober et al., 1985; Caramelli et al., 1998).

Episodic autobiographical memory and vSTM are related to language deficits in PWD. There has been an increasing interest in natural language processing and spontaneous speech in dementia (Lindsay et al., 2021), with researchers looking into their use as early markers (Luz et al., 2021). Language deficits can present at various levels in dementia, including word-finding, sentence comprehension, and discourse cohesion (Kempler and Goral, 2008). Previous studies in dementia mostly focused on syntactic or more ‘basic’ units in language, such as lexico-semantic changes (Szatloczki et al., 2015). In dementia, unlike aphasia or other brain conditions, language deficits caused by a focal brain damage is rare (Kempler and Goral, 2008). Discourse is a language unit whose organization supersedes any single words or sentences (Olness, 2006). It provides a multidimensional evaluation of various linguistic levels of spoken output (Filiou et al., 2019). Some studies have suggested that discourse production involves higher cognitive demands, it is more sensitive than other linguistic assessments, such as naming and verbal fluency, in distinguishing PWD from controls (e.g., Caramelli et al., 1998). In particular, memory plays a vital role in producing discourse (Caramelli et al., 1998; Dijkstra et al., 2004). Episodic memory is responsible for the retrieval of past information, especially in producing personal narratives (Caspari and Parkinson, 2000; Beltrami et al., 2018). vSTM, on the other hand, is required to store verbal information temporarily to continue the flow of spoken discourse (Brandão et al., 2009). On the other hand, Mueller et al. (2018), for example, provided a discussion of the pros and cons of using connected speech tasks, and cited at least one study that noted no advantage of picture description over naming and verbal fluency. Moreover, Gordon and Kindred (2011) explained that speakers have the option of selecting alternative words to compensate for word-retrieval impairments when producing a discourse, with a higher degree of flexibility to achieve coherence and cohesion. It, therefore, remains unclear whether discourse is a more sensitive task to reflect cognitive impairments.

The detailed relationship between memory and discourse remains elusive. Studies conducted in populations with neuro-communicative disorders have found the following: in traumatic brain injury, working memory (WM) was found to be correlated with syntactic complexities (Youse and Coelho, 2005) while vSTM was linked to informativeness and global coherence (Galetto et al., 2013); in aphasia, WM was associated with global coherence in story retell (Cahana-Amitay and Jenkins, 2018), but no such an association was observed in personal narratives (Rogalski et al., 2010). Limited research conducted in mild cognitive impairment (MCI) or dementia suggested that episodic memory is correlated positively with global coherence in autobiographical narratives in MCI (Seixas-Lima et al., 2020), and WM negatively correlated with the use of nominal references and pronouns in narratives and picture descriptions, respectively (Almor et al., 1999; March et al., 2009). Methodological variations likely contributed to the inconclusive findings, especially with the use of varied discourse tasks including narratives and picture descriptions (Hill et al., 2018). It is also worth highlighting that in cognitively healthy older adults, decline in performance on spoken oral discourse through story telling was found to significantly correlate with that of cognitive measures in memory and attention (Wright et al., 2011).

The above-mentioned studies were done predominantly in English speakers, although Chinese populations are the major drive in the continued growth in global dementia prevalence (Alzheimer's Disease International, 2013), with 9.5 million PWD currently residing in Hong Kong, Taiwan, and China (Wu et al., 2018). The relationship between language and memory may differ between English and Cantonese due to several factors, including the cultural background (Gutchess and Indeck, 2009), and linguistic structure and cognitive processes involved in using each language (Pennington and Ellis, 2000). In other words, with regard to language-memory relationships, results from studies in English might not be representative of those in Cantonese; this forms a strong argument for language diversity in studies on this issue. A handful of research has been done in Cantonese-speaking people with traumatic brain injury, revealing the positive correlation between attention, executive functions, visuo-spatial skills, and syntactic complexities (Kong et al., 2020; Lau et al., 2022). To our knowledge, no study has examined the relationship between memory and discourse production in native Cantonese-speaking PWD.

In this study, the relationship between memory and discourse production in Cantonese-speaking PWD is investigated, using discourse produced in both personal narrative and picture description. Our aim is to examine if discourse can be utilized clinically to inform methods of diagnosing dementia. Specifically, based on the High Level Language Hypothesis (Galetto et al., 2013), macrolinguistic deficits could be attributed to impaired conceptual organization of a narrative; it was therefore hypothesized that (1) there would be a positive association between episodic memory and global coherence of personal narrative. In addition, organizing a discourse requires a person to temporarily move forward or backward between mental sets, a crucial component in vSTM; it was therefore hypothesized that (2) vSTM would be correlated with informativeness of discourse production and empty speech. Moreover, which discourse measure(s) would best predict(s) memory deficits was explored.

## 2. Methods

### 2.1. Participants and discourse samples

Discourse samples were collected from 119 participants at baseline from a pilot study to investigate virtual delivery of non-pharmacological interventions to community-dwelling families living with dementia during COVID lockdown. Inclusion criteria of the study were a diagnosis of mild/moderate dementia as indicated in the referral and/or medical documentation and able to provide a joint consent with a family carer; exclusion criteria were inability to communicate and participate in interviews and intervention via a tablet computer, and severe visual or hearing impairment. Participants were users of local social programs (including community dementia care, aged care, and housing service users) recruited from service units in Hong Kong. After data screening, personal narratives of 49 participants and picture descriptions of 15 participants were excluded because of one of the following three problems: total words fewer than 40 [as transcripts of this length did not contain sufficient amount of content for a valid linguistic analysis (Saffran et al., 1989; Kong, 2022)], lack of discourse samples produced, or incomplete discourse task. A final 70 personal narratives and 104 picture description samples were included from 104 participants (see Table 1 for their demographic characteristics). *T*-test results indicated that the subgroup of 70 and the original group of 104 participants were not significantly different in terms of age [*t* (172) = 0.614, *p* = 0.423] and education [*t* (172) = −0.409, *p* = 0.434]. Chi-square results also revealed the two group were not significantly different in dementia severity [*χ*^2^ (2,174) = 0.415, *p* = 0.981].

**Table 1:** Demographic characteristics of participants (total *n* = 104).

	Number of participants (%)	
Characteristics	*N* = 104	Subgroup of *n* = 70 with personal narratives
Age		
60–69	6 (5.7%)	4 (5.7%)
70–79	27 (26.0%)	20 (28.6%)
80–89	59 (56.7%)	46 (65.7%)
90 or above	12 (11.5%)	6 (8.6%)
All	104 (100%); 81.8 ± 7.5, 63–98^*^	70 (100%); 81.1 ± 6.9, 64–97^*^
Gender		
Male	38 (36.5%)	28 (40%)
Female	66 (63.5%)	42 (60%)
Years of education		
0–3	38 (36.5%)	25 (35.7%)
4–6	27 (26.0%)	15 (21.4%)
7–9	8 (7.7%)	7 (10%)
10 or above	25 (24.0%)	19 (27.1%)
Unknown	6 (5.8%)	4 (5.7%)
All	104 (100%); 6.17 ± 5.1, 0–19^*^	70 (100%); 6.52 ± 5.4, 0–19^*^
Severity of dementia		
Suspected	10 (9.5%)	8 (11.4%)
Mild	47 (44.8%)	31 (44.3%)
Mild-to-moderate	12 (11.4%)	8 (11.4%)
Moderate	29 (27.6%)	19 (27.1%)
Unknown	7 (6.7%)	4 (5.7%)

*Data are presented in Mean ± SD, range.

### 2.2. Procedures

Discourse samples were collected by trained researchers following the Cantonese Aphasia Bank protocol (Kong and Law, 2019). For the personal narrative discourse, participants were asked a probing question “Tell me about the most joyful event in your life.” If there were no responses, general prompts (e.g., “how about traveling, or family events?”) were provided. For sequential picture description, they were asked to describe a sequential picture set (story of buying ice-cream) following the Main Concept Analysis (MCA) protocol (Kong, 2016), with proven sensitivity in distinguishing PWD from people with aphasia and controls (Kong et al., 2016). Participants were asked to tell a story portrayed in four picture cards pre-arranged in the correct order. If no relevant response was obtained, general prompts (e.g., “what is happening here?”) were provided. This sequential picture description elicited verbal output of temporally and causally related sequence of activities (Kong, 2022). The discourse samples were transcribed orthographically and then divided into T-units, defined as an independent clause with or without a subordinate clause (March et al., 2009; see Supplementary Table S1 for special cases of segmentation of T-units in Cantonese), for analysis.

Participants were assessed for their cognitive performance by trained researchers using the Hong Kong Montreal Cognitive Assessment (HK-MoCA) 5-min Protocol (Wong et al., 2015) and a Cantonese version of the Oxford Cognitive Screen-Plus (OCS-Plus; Demeyere et al., 2021). Demographic characteristics including age, gender, education, and dementia severity were collected through interviews with carers.

### 2.3. Measures

*Memory measures.* Episodic autobiographic memory was assessed following the protocol of Seixas-Lima et al. (2020). Each T-unit in a personal narrative was classified as episodic if it reflected re-experiencing of events specific to time and place, including happenings, spatial, temporal, and perceptual information and internal states, i.e., thoughts of feelings (Levine et al., 2002). The number of episodic details was tallied and divided by the number of T-units in the same narrative to compute the score of episodic memory. vSTM was assessed using the HK-MoCA, which generates immediate recall, delayed recall, delayed cued recall, and total recall scores, and OCS-Plus (Demeyere et al., 2021), which generates delayed recall, recognition recall, and total recall scores. A vSTM composite score was also computed for the seven measures from HK-MoCA and OCS-Plus. The raw scores of each measure were converted into Z-scores, and a weighted mean of these Z-scores was calculated, forming the final composite score.

*Discourse measures.* Global coherence, the linkage between the main topic and contents of individual utterances of the discourse (Wright et al., 2014), was assessed using a 4-point rating scale (Seixas-Lima et al., 2020). Each T-unit was rated from 0 to 3, based on the degree of propositional information relevant to the main topic. An average score was computed to represent global coherence for each sample (see Supplementary Tables 2 for specific scoring criteria).

Informativeness was evaluated using (a) MCA, (b) information rating of the Cantonese version of Western Aphasia Battery (CAB; Yiu, 1992), and (c) indices of empty speech. MCA measures presence and completeness of information in a discourse (Kong, 2022). For (a) MCA, six indices, including accurate and complete, accurate but incomplete, inaccurate, and absent concepts, overall main concept score, and ‘accurate and complete’ concepts per minute (Kong, 2009), were calculated for sequential picture description; MCA was not applied for personal narrative as the subjective nature of personal narrative precluded objective assessment of information accuracy and completeness. For (b) information rating of CAB, originally developed to assess connected speech in picture description, it was used to subjectively assess the informativeness of discourse production, with reference to number of correctly named items. For (c) empty speech, a main characteristic of PWD, it refers to reduced informative content and lack of references in connected speech (Nicholas et al., 1985). We applied six indices (see Supplementary Table S3 for details) to both personal narrative and picture description: percentage of pronouns adopted from Almor et al. (1999); and pronouns without antecedents, deictic terms, repetitions, empty phrases, and comments derived from Nicholas et al. (1985). For the last four indices, raw counts were divided by the number of T-units in each discourse sample to obtain a ratio.

### 2.4. Inter- and intra-rater reliability

A second independent examiner, who was a speech-language pathologist trainee (i.e., similar to the second author, or first examiner, in terms of background/experience) and received training on calculating the measures, reviewed 10 personal narratives and 15 picture descriptions (15% of samples) that were randomly selected. The same set of samples were reviewed by the second author two months after initial analyses. The inter-rater and intra-rater reliability were measured using Intraclass Correlation Coefficient. Both reliability measures were high overall, with most ICC results reaching levels of good to excellent (Koo and Li, 2016; see Supplementary Table S4). A discrepancy was only found in inter-rater reliability for indices ‘accurate but incomplete’ and ‘inaccurate’ concepts, where ‘accurate but incomplete’ was rated as ‘inaccurate’, and ‘inaccurate’ was rated as ‘absent’; this was similar to previous report of decreased reliability when more than one incomplete or inaccurate concept was present in a PWD’s description (Kong et al., 2016).

### 2.5. Statistical analysis

Since the data were not normally distributed, a non-parametric test of Spearman’s Rank was conducted to explore the correlations between memory and discourse measures. An adjustment of significance level was done using Bonferroni’s method due to multiple comparisons of memory measures (0.05/3 or 0.0167). Since the number of variables under consideration was large, a forward stepwise analysis was utilized in SPSS (George and Mallery, 2019), with the vSTM composite score and episodic memory score being the dependent variables, and all discourse measures as independent variables in the regression model. Given the range of age and education, these variables were included in the regression analyses. This forward selection started with a null model (with no predictors) and proceeds to add variables one at a time, and so unlike backward selection, it does not have to consider the full model that which would include all the predictors.

## 3. Results

The descriptive statistics of all memory and discourse measures can be found in Supplementary Table S5. Tables 2, 3 summarize the correlations between various memory and discourse measures in the personal narrative and picture description tasks, respectively.

**Table 2:** Correlation between memory and discourse measures in personal narratives.

		Global coherence	Use of pronoun (%)	Deictic term	Repetition	Pronouns without antecedent (%)	Empty phrase	Comment
Episodic autobiographical memory		0.772***	−0.071	−0.159	0.004	−0.217	−0.221	−0.221
HK-MoCA	Immediate recall	0.125	0.092	−0.094	−0.296*	−0.080	0.037	0.201
	Delayed recall	0.092	0.163	−0.100	−0.010	−0.098	0.057	0.120
	Cued delayed recall	0.363**	−0.039	−0.004	0.013	−0.200	0.013	0.001
	Total recall	0.282	0.021	0.029	−0.017	−0.174	0.066	0.036
	Total score	0.332**	0.042	−0.018	−0.126	−0.108	−0.032	0.055
OCS-Plus	Delayed recall	0.293	−0.035	−0.014	0.032	−0.056	0.108	0.138
	Recognition recall	0.085	−0.014	−0.070	0.058	−0.160	−0.254	−0.146
	Total recall	0.237	−0.027	−0.065	0.086	−0.163	−0.139	−0.022

The significant correlations were bolded. *p < 0.0167; **p < 0.01; ***p < 0.001.

**Table 3:** Correlations between memory and discourse measures in picture descriptions.

		Global coherence	Use of pronoun (%)	Deictic term	Repetition	Pronouns without antecedent (%)	Empty phrase	Comment	MC score	CAB info.
Episodic autobiographical memory		0.478***	0.109	0.208	−0.076	−0.253	−0.009	−0.356**	0.454***	0.501**
HK-MoCA	Immediate recall	0.129	−0.081	−0.031	−0.133	−0.177	−0.208	−0.104	0.125	0.139
	Delayed recall	0.107	0.033	−0.011	−0.128	0.069	−0.107	−0.038	0.152	0.197
	Cued delayed recall	0.366***	−0.169	−0.221	−0.178	−0.134	−0.285**	−0.156	0.416***	0.437***
	Total recall	0.306**	−0.129	−0.170	−0.178	−0.061	−0.262**	−0.110	0.348***	0.393***
	Total score	0.361***	−0.058	−0.095	−0.295**	−0.077	−0.297**	−0.109	0.443***	0.481***
OCS	Delayed recall	0.281**	−0.119	−0.129	−0.151	0.008	0.025	−0.065	0.337***	0.293**
	Recognition recall	0.283**	−0.050	−0.030	−0.172	−0.189	−0.158	−0.100	0.218	0.259**
	Total recall	0.382***	−0.101	−0.085	−0.238*	−0.128	−0.113	−0.106	0.352***	0.358***

MC score, main concept score; CAB info. = Cantonese Aphasia Battery information rating. The significant correlations were bolded.

*p < 0.0167; **p < 0.01, ***p < 0.001.

### 3.1. Exploratory correlational analysis

In the personal narrative task, global coherence positively correlated with episodic autobiographical memory, HK-MoCA cued delayed and total recall, OCS-Plus delayed and total recall. Among all, episodic autobiographical memory and global coherence yielded the highest correlation coefficient (*r = 0*.772*, p < 0*.001). For empty speech indices, use of repetitions negatively correlated with HK-MoCA immediate recall (*r* = 0.296, *p* < 0.0167).

For the sequential picture description task, global coherence significantly correlated with episodic memory (*r = 0*.478*, p < 0*.001) and vSTM measures in HK-MoCA (e.g., cued delay recall: *r = 0*.366*, p < 0*.001) and OCS-Plus (e.g., delayed recall: *r = 0*.281*, p < 0*.01). Negative correlations were found between memory and all empty speech indices (except for percentage of pronouns), although these correlations were relatively weak, with most of the Spearman *r* < 0.30. Significant correlations were found between most memory tests and the MC score. Information rating of CAB also positively correlated with all memory measures, except for immediate and delayed recall in HK-MoCA.

### 3.2. Regression analysis

All discourse measures were entered against episodic autobiographical memory and vSTM composite score (Tables 4, 5). For the model of episodic autobiographical memory, global coherence of both genres and use of deictic terms in picture description were significant predictors, accounting for a total variance of 70.0% [*F* (3,64) = 47.499, *p* < 0.001]. It is worth noting that global coherence in personal narrative alone accounted for 61% variance of episodic autobiographical memory. For the model of vSTM composite score, the regression analysis was significant [*F* (1,67) = 19.273, *p* < 0.001], with information rating of CAB being the only discourse predictor of vSTM. It accounted for 48.4% of the total variance. The variables of age and education did not yield any significant contributions to the regression models.

**Table 4:** Stepwise regression of episodic autobiographical memory as dependent variable.

Predictor	*B*	SE B	*β*	*t*	*p*
Step 1					
Constant	−0.005	0.078		−0.062	0.951
PN GCR	0.323	0.032	0.789	10.205	< 0.001
Step 2					
Constant	−0.084	0.080		−1.045	0.300
PN GCR	0.325	0.030	0.794	10.762	< 0.001
SPD deictic terms	0.171	0.064	0.199	2.691	0.009
Step 3					
Constant	−0.144	0.079		−1.826	0.073
PN GCR	0.282	0.033	0.690	8.666	< 0.001
SPD deictic terms	0.213	0.062	0.248	3.426	0.001
SPD GCR	0.086	0.031	0.226	2.771	0.007

PN GCR = global coherence of personal narratives; SPD = sequential picture description.

Adjusted *R*^2^ = 0.617 for step 1 (*p* < 0.001); ΔR^2^ = 0.663 for step 2 (*p* < 0.05); Δ*R*^2^ = 0.685 for step 3 (*p* < 0.05).

**Table 5:** Stepwise regression of vSTM composite score as dependent variable.

Predictor	*B*	SE B	*β*	*t*	*p*
Step 1					
Constant	−0.796	0.201		−3.965	< 0.001
CAB information	0.129	0.029	0.484	4.390	< 0.001

CAB, Cantonese Aphasia Battery.

Adjusted *R*^2^ = 0.234 for step 1 (*p* < 0.001).

## 4. Discussion

This study provided, to our knowledge, the first evidence of the close relationship between discourse performance and memory in a sizable Chinese sample of PWD with standardized discourse measures. We noted a particularly strong positive relationship between episodic autobiographical global coherence, which echoed an earlier study in people with MCI (Seixas-Lima et al., 2020). These findings showed an important role of discourse as part of the clinical presentation in neurocognitive disorders that affect global cognition, through its association with episodic memory and vSTM.

Our finding that global coherence is correlated with vSTM measures is in line with previous studies (Brandão et al., 2009; Kim et al., 2019). The relationship between informativeness in picture description (as reflected by the main concept performance) and vSTM confirmed previous reports in AD and traumatic brain injury (Brandão et al., 2009; Galetto et al., 2013). It can be interpreted based on reports investigating neural correlates of vSTM and language production. Overlapping areas of activation (left inferior frontal and left posterior temporal areas) between vSTM and language production have been widely reported (Melrose et al., 2009; Peters et al., 2009; Koenigs et al., 2011). The association between informativeness in picture description and episodic memory observed in this study is interesting: as visual stimuli were provided, we expected minimal involvement of episodic memory in the picture description task. A plausible explanation is the relationship between long-term memory, the representational basis of vSTM (Cameron et al., 2005), and vSTM. When vSTM is engaged, long-term memory is activated to help with the maintenance of semantically related information. Better long-term memory capacity, including episodic memory, could help enhance vSTM during discourse production.

The negative correlation between empty speech (i.e., deictic terms, repetitions, empty phrases) and vSTM is worth noting. Although PWD have been shown to produced significantly more deictic terms, repetitions, and empty phrases than controls (Kong et al., 2016), no quantitative studies examining their relationships with vSTM have been reported. Empty speech in PWD was suggested to stem from both linguistic and cognitive disturbances, such as memory and attention (Carlomagno et al., 2005; Kong et al., 2016). Our finding can be understood based on two key theories of vSTM, output interference and response suppression, which suggest that recalling of an item interferes with the uncalled ones that needs to be suppressed, otherwise it would continue to be activated (Lewandowsky, 2008). As empty speech in PWD is often manifested by occurrence of deictic terms, repetitions, and empty phrases, deficits in vSTM would further reinforce these frequently activated items (which would be overused in discourse). However, it should also be noted that although the association were statistically significant, these correlations were relatively weak. This might be related to the uneven distribution of severity level of PWD. More than half of our participants (65.7%) had a severity of mild-to-moderate or below, while only around a quarter of them (27.6%) was diagnosed with moderate dementia. Previous studies showed that empty speech was more likely to manifest in middle or late stage of dementia (March et al., 2009; Forbes-McKay et al., 2013; Kong et al., 2016). Therefore, the memory impairment of our participants might not be severe enough for empty speech to manifest.

The finding that global coherence is the best predictor of episodic autobiographical global coherence in personal narrative is significant yet unsurprising, considering the essential functions of episodic memory in recalling specific time, location, and thoughts (El Haj et al., 2015) to maintain a coherent personal narrative. While both episodic autobiographical memory and semantic autobiographical memory (i.e., “personal semantics” or semantic knowledge about oneself, see Conway, 2005) changes are clinical hallmarks in dementia, with a recent study showing their higher sensitivity over standard neurocognitive tests in cognitively unimpaired people with increased genetic risk (APOE4 carriers) (Grilli et al., 2021), episodic autobiographic memory is possibly a more important marker and intervention target: its differential impairment is linked to underlying disease pathology in different dementia types including AD and frontotemporal dementia (Irish et al., 2011). Its role in supporting re-experiencing of the past (Irish et al., 2018) is theoretically central to interventions targeting personhood in dementia, such as cognitive stimulation therapy (CST) and reminiscence therapy. These interventions typically involve conversations on autobiographical topics; their mechanisms of action on cognitive outcome are unclear, although in CST the cognitive enhancement effects (Woods et al., 2012) may be linked to language use (Spector et al., 2010; Lobbia et al., 2019) and brain networks responsible for episodic memory retrieval and mental self-representation (Liu et al., 2021). Our finding that global coherence explained over half of the variance in episodic autobiographic memory provided further insight into how conversations revolving around personal experience may be associated with episodic memory and a cohesive sense of self over time (Strikwerda-Brown et al., 2019) as an intervention target in dementia. Potential strategies may include spaced retrieval and post-sentential training, to support PWD’s memory loading in spoken discourse production (Brush and Camp, 1998).

Information rating of CAB was the only significant predictor of vSTM in our regression analysis. This rating scale was originally designed for people with aphasia instead of PWD (Yiu, 1992). Since it is an overall scoring of relevant content, including any naming and descriptions, one may argue that this scale has a broader scope of scoring than other content-based measures, such as MCA, which might explain why it could be more sensitive in predicting vSTM. This study offers preliminary evidence that information rating might be clinically useful in understanding discourse production in PWD. Future studies might focus on how to adjust the scoring criteria of the rating so it can be better adapted for PWD.

Our overall findings are in line with previous literature which demonstrated a close relationship between global coherence and memory measures (Drummond et al., 2015; Kim et al., 2019). In addition, the close link between deictic elements of a language (which contain limited meaning in sentences) and memory seemed to also help the formation of utterances to a particular time, place, speaker, or discourse context (Brewer and Harris, 1974). The current study in a Chinese sample adds to a growing literature of speakers of different languages (Fleming and Harris, 2008; March et al., 2009; Kim et al., 2019), including Western studies showing significant associations between working memory and discourse measures (Almor et al., 1999; Youse and Coelho, 2005; Cahana-Amitay and Jenkins, 2018). It contributes to the knowledge base supporting the emerging research methods of natural language processing and automated speech analysis in dementia, which is increasingly noted with its potential link to clinician observations (Yeung et al., 2021). This being said, however, the potential values of discourse measures as markers of memory ability needs to be further evaluated, as our cross-sectional findings are essentially preliminary. From the present findings, it may be difficult to see how the discourse measures (which are indirectly and sometimes weakly related to the memory measures, not to mention being more labor-intensive to collect and process) can provide an advantage over the memory measures themselves. We argue that discourse measures and other memory measures in dementia are likely complementary: discourse data are in general easier to conduct (simple training not requiring specific qualifications; less intimidating among older people with lower education/cognitive impairment to engage in a chat than a test; can be easier conducted remotely) but more challenging to rate, although the rapid development in machine learning (e.g., see Lindsay et al., 2021) suggests this challenge may be temporary. As a potential screening tool for people with suspected dementia, the ease of data collection is particularly important, considering the many barriers (e.g., stigma and fear) currently exist in dementia help-seeking. In future studies, apart from the cognitive screening tools (HK-MoCA and OCS-Plus) used in this study, more refined cognitive tests and other functional outcomes should be included in longitudinal studies to understand its performance as a simple memory marker and potential predictor of illness severity in dementia.

Finally, there were several limitations in the present investigation. First, the length of the included narrative samples was relatively short. The mean length of narrative and picture description samples was 70 and 80 characters, respectively. Although there was no reported word recommendation on Cantonese discourse samples, previous studies recognized the problems of insufficient length of discourse samples, resulting in insignificant findings of correlation analysis (Mueller et al., 2018). This might explain some of the insignificant correlations observed in the personal narrative task. For English-speaking PWD, it has been suggested a mean length of 100 words for discourse samples (Fraser et al., 2016) might act as a reference for future studies. Second, due to a lack of participants with middle or late stage of dementia, the current discourse samples cannot fully represent discourse produced with the cognitive deficits in later stages of dementia. Lastly, due to the cross-sectional nature of this study, no direction of the relationship can be inferred. One may also argue that the CAB information rating scale might contain a confound as it includes autobiographical questions, making it difficult to draw a clear conclusion about the relative importance of the predictors. This study nevertheless provided detailed discourse measures for identifying important markers/intervention targets for understanding the link between episodic autobiographical memory, vSTM, and various discourse measures. Future longitudinal or experimental research can examine the direction of relationships to inform prediction model and intervention design.

## Data availability statement

The original contributions presented in the study are included in the article/Supplementary material, further inquiries can be directed to the corresponding author/s.

## Ethics statement

The studies involving humans were approved by the Human Research Ethics Committee of The University of Hong Kong. The studies were conducted in accordance with the local legislation and institutional requirements. The participants provided their written informed consent to participate in this study.

## Author contributions

AK and RC contributed to the conception and design of the study, and wrote the first draft of the manuscript. GW, JC, and RD contributed to the conception of the study and were responsible for data collection. AS contributed to the conception of the study. JC has gone through the official certification process of HK-MoCA and obtained appropriate permission the copyright holders of HK-MoCA to use this scale. All authors contributed to manuscript revision, read, and approved the submitted version.
