# Supplementary material for: Spoken discourse in episodic autobiographical and verbal short-term memory in Chinese people with dementia: the roles of global coherence and informativeness
Source: Front Psychol. 2023 Oct 31;14:1124477. doi: 10.3389/fpsyg.2023.1124477 (PMC10643863; doi:10.3389/fpsyg.2023.1124477)
Supplement: Supplementary file 1 [file Table_1.DOCX]

Supplementary Material

Spoken discourse in episodic autobiographical and verbal short-term memory in Chinese people with dementia: The roles of global coherence and informativeness

Anthony Pak-Hin Kong*, Ryan Tsz Him Cheung, Gloria H.Y. Wong, Jacky C.P. Choy, Ruizhi Dai, Aimee Spector

*** Correspondence:** Anthony Pak-Hin Kong: [akong@hku.hk](mailto:akong@hku.hk)

Supplementary Table 1. *Sentence structures that are common in Cantonese but not English*

| Sentence structures | Examples |
| --- | --- |
| Right dislocation | 好      幸福       我     覺得  Very  blessed     I      think  I am very blessed. |
| Omission of subjects | 喺   香港           考      呢個           西               醫             試  in  Hong Kong   take       this       Western     Medicine     Exam  I took this medical exam in Hong Kong |
| Topicalization | 日本     就       好似        未         去       過  Japan   then     seem     not yet     go     (particle marking perfect tense)  It seems that I have not been to Japan |

Supplementary Table 2. *Global Coherence Rating Scale and Score Computation*

| Score | Criteria | Example: A trip to USA |
| --- | --- | --- |
| 3  (No. of score 3=a) | Elements of the semantic frame given as a topic of discourse, i.e. information about facts, component states, actions or events directly related to the topic | 「不過我唔識路，要朋友帶」 |
| 2  (No. of score 2=b) | Relevant implications for the topic/ frame (necessary or probable conditions and consequences, i.e. what happened before or after an event), or a discourse cue supporting the discourse (such as a “yes” or “no” answer to a direct question). | 「自己未見識過嘅就見識下囉」 |
| 1  (No. of score 1= c) | Elements which do not belong to the semantic frame nor are relevant for the topic, or repeated information | 「有嘅，但係不過我唔識講比你聽喎」 |
| 0  (No. of score 0= d) | A detail which carries no propositional information (such as fillers or rhetorical questions) | 「係啊係啊」  「你真係好人啊」 |
| Computation: | 3xa+2xb+1xc+0xd/ total no. of T-units | |

*Note.* Adapted from Seixas-Lima et al. (2020).

Supplementary Table 3 *Operationalization of empty speech indices*

| **Indices** | **Operationalization** |
| --- | --- |
| **Percentage of pronouns** | Pronouns include personal pronouns (我、你、自己、我哋、你哋、佢、佢哋) (I, you, myself, we, you, him/her, them) and demonstrative pronouns (呢個、嗰個、呢啲、嗰啲) (this, that, these, those). Nominal references include all noun phrases. The number of pronouns was tallied and divided by the total number of nominal references to obtain a percentage. |
| **Pronouns without antecedents** | A pronoun did not have an antecedent if it was not referring to any preceding definite nouns. Exceptions were “我” (I) and “你” (you) referring to the participants themselves or the interviewer. The number of pronouns without antecedents was tallied and divided by the total number of pronouns. |
| **Deictic terms** | Deictic terms included “呢個、嗰個、呢啲、嗰啲、呢度、嗰度” (this, that, these, those, here and there). |
| **Repetitions** | Repetitions were counted when a character, word, or phrase was repeated within the same T-unit. |
| **Empty phrases** | Phrases that contributed no meaning to the discourse were regarded as empty phrases, such as “即係呢、好似係” (so it means, it is like). |
| **Comments** | Utterances addressing the nature, instead of content, of the task were regarded as comments, such as “好難喎、我唔識喎” (it is difficulty, I do not know). |

Supplementary Table 4 *Inter-rater and intra-rater reliability measures*

| Measures | Inter-rater reliability | Confidence Interval | Intra-rater reliability | Confidence Interval |
| --- | --- | --- | --- | --- |
| PN Episodic Autobiographical Retrieval | 0.958** | 0.839-0.989 | 0.997** | 0.987-0.999 |
| PN Global Coherence | 0.934** | 0.767-0.985 | 1** | 0.998-1.00 |
| PN % of pronouns | 0.933** | 0.967-0.998 | 0.997** | 0.988-0.999 |
| PN pronouns without antecedents | 0.952** | 0.813-0.988 | 1 | / |
| PN deictic terms | 0.841* | 0.328-0.961 | 0.971** | 0.889-0.993 |
| PN repetitions | 1 | / | 0.973** | 0.899-0.993 |
| PN empty phrases | 0.928** | 0.715-0.982 | 0.938** | 0.765-0.984 |
| PN comments | / | / | / | / |
| SPD Global Coherence | 0.991** | 0.972-0.997 | 0.984** | 0.952-0.995 |
| SPD % of pronouns | 0.862** | 0.601-0.953 | 1** | 0.999-1.00 |
| SPD pronouns without antecedents | 0.98** | 0.943-0.993 | 1 | / |
| SPD deictic terms | 0.805** | 0.30 -0.946 | 0.996** | 0.987-0.999 |
| SPD repetitions | 1 | / | 1 | / |
| SPD MCA AC | 0.994** | 0.982-0.998 | 0.897** | 0.704-0.965 |
| SPD MCA AI | 0.335 | -0.644-0.76 | 0.936** | 0.812-0.978 |
| SPD MCA IN | 0.547 | -0.239-0.843 | 0.907** | 0.730-0.968 |
| SPD MCA AB | 0.917** | 0.753-0.972 | 1 | / |
| SPD MCA MC Score | 0.958** | 0.876-0.986 | 0.993** | 0.981-0.998 |
| SPD MCA MC per Minute | 0.996** | 0.988-0.999 | 0.997** | 0.992-0.999 |
| SPD Information rating of CAB | 0.915** | 0.744-0.971 | 0.986** | 0.958-0.995 |

*Note.* PN= Personal narrative; SPD= Sequential Picture Description. * *p* < 0.01; ** *p* < 0.001.

Supplementary Table 5. *Descriptive statistics of memory and discourse measures*

| Measures | | N | Mean | SD | Range |
| --- | --- | --- | --- | --- | --- |
| Episodic autobiographical memory | | 70 | .76 | .29 | 0-1.0 |
| MoCA | Total score | 104 | 13.29 | 6.12 | .5 – 27.0 |
|  | Immediate recall | 104 | 2.35 | 1.48 | 0-5.0 |
|  | Delayed recall | 104 | .92 | 1.51 | 0-5.0 |
|  | Cued delayed recall | 104 | 2.94 | 1.72 | 0-5.0 |
|  | Total recall | 104 | 3.87 | 2.81 | 0-10.0 |
| OCS-Plus | Delayed recall | 104 | .28 | .830 | 0-4.0 |
|  | Recognition recall | 104 | 2.27 | 1.13 | 0-5.0 |
|  | Total recall | 104 | 2.55 | 1.31 | 0-5.0 |
| Personal narrative | Global Coherence Rating | 70 | 2.39 | 0.70 | .48-3.0 |
|  | % of pronouns | 70 | .28 | .21 | 0-1.0 |
|  | Deictic terms | 70 | .10 | .15 | 0-.50 |
|  | Repetitions | 70 | .04 | .10 | 0-.60 |
|  | Pronouns without antecedents | 70 | .11 | .25 | 0-1.0 |
|  | Empty phrases | 70 | .04 | .13 | 0-.90 |
|  | Comments | 70 | .01 | .03 | 0-.20 |
| Picture description | Global Coherence Rating | 104 | 1.58 | 0.81 | 0-3.0 |
|  | % of pronouns | 104 | .38 | .40 | 0-4.0 |
|  | Deictic terms | 104 | .39 | .33 | 0-1.40 |
|  | Repetitions | 104 | .13 | .22 | 0-1.50 |
|  | Pronouns without antecedents | 104 | .49 | .40 | 0-1.0 |
|  | Empty phrases | 104 | .06 | .18 | 0-1.17 |
|  | Comments | 104 | .17 | .20 | 0-.83 |
|  | MCA AC | 104 | .57 | 1.10 | 0-5 |
|  | MCA AI | 104 | .43 | .69 | 0-3 |
|  | MCA IN | 104 | .43 | .67 | 0-3 |
|  | MCA AB | 104 | 7.57 | 1.50 | 3-9 |
|  | MCA MC Score | 104 | 3.00 | 3.85 | 0-17 |
|  | MCA MC per Minute | 104 | .65 | 1.57 | 0-10.6 |
| CAB Information Rating | | 104 | 5.82 | 2.48 | 0-10 |

*Note.* CAB = Cantonese Aphasia Battery
